# Supplementary material for: LTA4H extensively associates with mRNAs and lncRNAs indicative of its novel regulatory targets
Source: PeerJ. 2023 Mar 10;11:e14875. doi: 10.7717/peerj.14875 (PMC10010175; doi:10.7717/peerj.14875)
Supplement: File S2 [file peerj-11-14875-s005.zip › Raw data files/Reference_genome/Length_distribution_across_Genomic_Regions.rtf]

Region_Type	length
CDS	32481
noncoding	31629
intron	1403490
intergenic	1705980
three_prime_UTR	34710
five_prime_UTR	8193
